# Phylogeny of European Bat Lyssavirus 1 in *Eptesicus isabellinus* Bats, Spain

**DOI:** 10.3201/eid1703100894

**Published:** 2011-03

**Authors:** Sonia Vázquez-Morón, Javier Juste, Carlos Ibáñez, José M. Berciano, Juan E. Echevarría

**Affiliations:** Author affiliations: Instituto de Salud Carlos III, Majadahonda, Madrid, Spain (S. Vázquez-Morón, J.M. Berciano, J.E. Echevarría);; Centro de Investigación Biomédica de Epidemiología y Salud Pública, Barcelona, Spain (S. Vázquez-Morón, J.E. Echevarría);; Consejo Superior de Investigaciones Científicas Estación Biológica de Doñana, Seville, Spain (J. Juste, C. Ibáñez)

**Keywords:** Lyssavirus, bats, phylogeny, rabies, EBLV-1, Eptesicus isabellinus, viruses, Spain, dispatch

## Abstract

To better understand the epidemiology of European bat lyssavirus 1 (EBLV-1) in Europe, we phylogenetically characterized *Lyssavirus* from *Eptesicus isabellinus* bats in Spain. An independent cluster of EBLV-1 possibly resulted from geographic isolation and association with a different reservoir from other European strains. EBLV-1 phylogeny is complex and probably associated with host evolutionary history.

The genus *Lyssavirus* comprises 3 species that can infect bats in Europe: *European bat lyssavirus 1* (EBLV-1), *European bat lyssavirus 2*, and *West-Caucasian bat virus* (*1*,*2*). Most lyssavirus-infected bats have been found in north-central Europe (Germany, the Netherlands, Denmark, Poland, and France); of these, >95% were serotine bats (*Eptesicus serotinus)* infected by EBLV-1 (*3*–*5*). EBLV-1 in other bat species has rarely been described (*3*,*6*). EBLV-1–infected bats become increasingly scarce from north to south in Europe, and no cases in northern Spain or Italy have been reported. The same trend has been consistently found within Germany (*3*) except for an artifact that arose from varied surveillance intensity among different countries. However, several infected serotine bats in southern Spain have been reported (*7*). These bats have been assigned to the species *E. isabellinus*, which has closely related populations on the African side of the Gibraltar Strait (*8*). This species is strongly divergent from *E. serotinus* bats (>16% of cytochrome b gene) in the northern Iberian Peninsula (*9*). In Spain, the distribution of EBLV-1 cases in bats apparently coincides with the distribution of *E. isabellinus* bats; 10 cases of human exposure after contact with infected bats have been reported; each was associated with *E. isabellinus* bats.

Two subtypes have been proposed for EBLV-1: EBLV-1a, which extends from the Netherlands to Russia in a west–east axis, and EBLV-1b, which includes strains that extend south through France and the Netherlands and the only 2 published strains from Iberia (*1*). We phylogenetically characterized EBLV-1 strains associated with *E. isabellinus* bats, a reservoir in the Iberian Peninsula that differs from *E. serotinus* bats.

## The Study

We sequenced 12 bat brains positive for *Lyssavirus* antigen detected by immunofluorescence and reverse transcription–PCR (RT-PCR) as described (*10*). All viruses were identified as EBLV-1. For phylogenetic analyses, the 400-bp 5′ variable extreme of the nucleoprotein gene of these EBLV-1 strains was amplified by specific EBLV-1 nested RT-PCR and sequenced by using the following primers: SEQVAR1F 5′-_1_ACGCTTAACAACCAGATCAAAG_22_-3′, SEQVAR2F 5′-_51_AAAAATGTAACACYYCTACA_70_-3′, EBLVSEQVAR1R 5′-_596_CAGTCTCAAAGATCTGTTCCAT_575_-3′, and EBLVSEQVAR2R 5′-_552_TAGTTCCCAGTATTCTGTCC_533_-3′.

**Figure 1:**
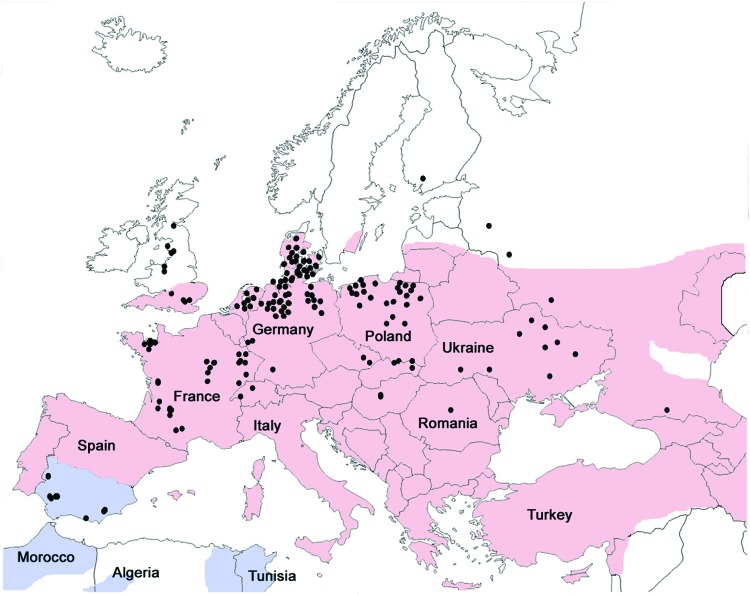
European bat lyssavirus 1 (EBLV-1) phylogenetic reconstruction based on the first 400 bp of the nucleoprotein gene. The tree was obtained by Bayesian inference run for 10^7^ generations; trees were sampled every 100 generations. The first 25% of trees were excluded from the analysis as burn-in. Black numbers indicate posterior probabilities. Bootstrap supports after 1,000 replicates for each node are also shown for maximum-parsimony (green numbers) and maximum-likelihood (blue numbers) analyses. Net *p*-distance values (as percentages) between groups are indicated by arrows. A parsimony-based network is presented for each major lineage; sizes of yellow circles are proportional to the number of individuals sharing a given haplotype, and reconstructed haplotypes (median vectors) are shown in red. DUVV, Duvenhage virus.

All rabies-positive serotine bats came from southern Spain (Huelva, Seville, Murcia, and Badajoz) and were molecularly identified as *E. isabellinus* (*8*). An alignment was performed by using ClustalX (www.clustal.org) to combine the obtained sequences and other available EBLV-1 sequences from GenBank, including a Duvenhage virus used as the outgroup (Table A1). Before conducting further analyses, we used jModelTest (http://darwin.uvigo.es/software/jmodeltest.html) to select the best fitting substitution model for our sequences according to the corrected Akaike information criterion. Maximum-likelihood phylogenies were reconstructed by using PHYML (http://atgc.lirmm.fr/phyml) software and by using a generalized time-reversible model and the γ parameter estimated in the analyses. Maximum-parsimony analyses were conducted by using PAUP* 4.0b10 (http://paup.csit.fsu.edu/) weighting transversions 15× according to the transitions/transversion ratio estimated in the jModelTest analyses. Confidence in the topologies for the maximum-likelihood and the maximum-parsimony analyses was established with 1,000 bootstrap replicates. A Bayesian phylogenetic inference was obtained by using MrBayes version 3.1 (http://mrbayes.csit/fsu.edu/) with random starting trees without constraints. Two simultaneous runs of 10^7^ generations were conducted, each with 4 Markov chains, and the trees were sampled every 100 generations. Net p-distances between groups were calculated by using MEGA4 (www.megasoftware.net) (Figure 1).

The genetic structure and relationships between haplotypes were examined within the main lineages through a parsimony-based network built with a median-joining algorithm implemented in the Network 4.5.1 program (*11*). To evaluate and compare genetic variability and polymorphism among lineages, we estimated the number of haplotypes, mutations, and segregating sites as well as haplotype diversity and nucleotide diversity by using DNAsp version 4.5 (*12*) for the major clades (Table). Finally, to investigate population dynamics across lineages, the Fu F*s* and Tajima D statistics were calculated (Table). These 2 statistics are considered to be the most powerful tests for detecting expansion events (*13*).

**Table:** . Genetic diversity statistics for EBLV-1*

Population	n	S	Eta	Hap	Hd	VarHd	Pi	ThetaNuc	k	Tajima D	Fu Fs
EBLV-1a	52	45	48	26	0.836	0.00267	0.00664	0.02656	2.6546	–2.5693 (0.00000)	–21.676 (0.00000)
EBLV-1b	25	35	35	18	0.970	0.00038	0.02202	0-02317	8.8067	–0.1885 (0.48000)	–4.555 (0.05100)
EBLV-1Spain	13	9	9	7	0.795	0.01191	0.00538	0.00725	2.1538	–1.0138 (0.18100)	–2.067 (0.06143)

*EBLV, European bat lyssavirus; n, no. sequences; S, no. segregating sites; Eta, no. mutations; Hap, no. haplotypes; Hd, haplotype diversity; VarHd, haplotype variance; Pi, nucleotide diversity; ThetaNuc, estimated population mutation rate per site; k, average no. nucleotide differences; and neutrality tests (Tajima D and Fu Fs).

## Conclusions

**Figure 2:**
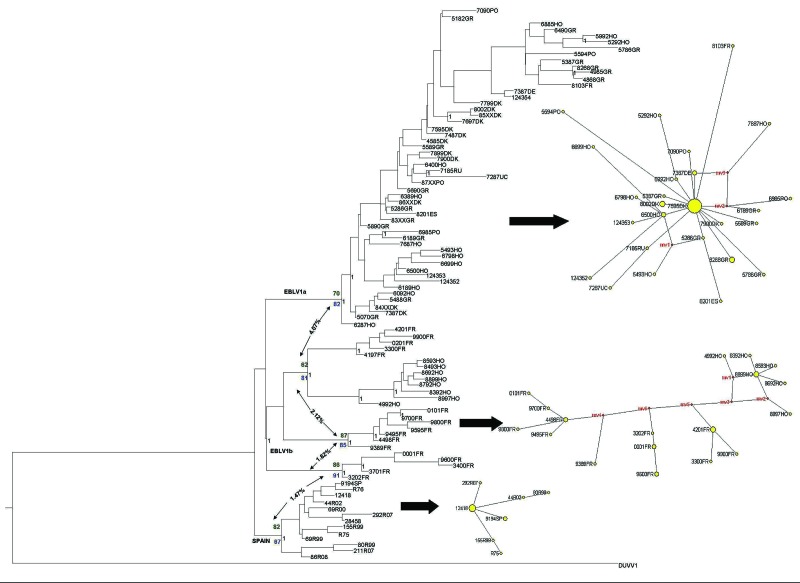
Geographic distribution of *Eptesicus serotinus* bats (red), *E. isabellinus* bats (blue), and cases of rabies in bats (green dots), Europe, 1990–2009. Obtained from Rabies Bulletin Europe (www.who-rabies-bulletin.org/).

All phylogenetic analyses, regardless of the reconstruction criterion used, formed a monophyletic cluster of the EBLV-1 strains from Spain (only the Bayesian inference reconstruction is shown). The Bayesian inference, maximum-likelihood, and maximum-parsimony analyses identified the cluster from Spain and EBLV-1a and EBLV-1b as being monophyletic (Figure 1), although only maximum-likelihood and maximum-parsimony analyses suggested a closer relationship between EBLV-1a and the cluster from Spain. The genetic differentiation of the EBLV-1 strains from the Iberian Peninsula matches their association with another bat species (Figure 2), which suggests that the host bat’s evolutionary history plays a major role in EBLV-1 molecular epidemiology, as has been proposed for rabies virus in bats in North America (*14*).

The low genetic diversity and the Fu F*s* and Tajima D statistics (Table) all suggest rapid population expansion of EBLV-1a, which is consistent with the star-like structure of the network for this lineage (Figure 1). Conversely, haplotype and nucleotide diversity descriptors (Table) have the highest values for EBLV-1b and a complex network structure with differentiated subnetworks. All these elements indicate that this lineage has a complex evolutionary history. The lineage from Spain also has low diversity and a star-shaped network, but neutral evolution cannot be rejected on the basis of the F*s* and D statistics. Net distances are similar within and between lineages, except for EBLV-1a, which is slightly more differentiated (Figure 1). Consequently, the suggested EBLV-1 expansion from Spain into Europe (*15*) is not supported by our results, which record the highest variability and most complex phylogenetic structure for France and the Netherlands (Figure 1). This complex structure suggests either a longer evolutionary history in these areas or a recent contact of distinct bat lineages in this zone.

The results of this study show that the strains from Spain do not belong to subtype 1b because of their association with a different reservoir (*E. isabellinus* bats*)*. Moreover, what is currently considered to be EBLV-1b seems to include at least 4 lineages that are more genetically diverse and have a complex history. EBLV-1a, however, has low genetic diversity despite its extensive geographic distribution, suggesting a relatively recent and successful expansion of this lineage. These results call into question the current classification of EBLV-1 into 2 single subtypes. To provide a better understanding of EBLV-1 molecular epidemiology in Europe, additional studies that consider different genes should be conducted and the current classification should be revised accordingly.

## Appendix

**Table A1:** EBLV-1 strains used in study of EBLV phylogeny in bats, Spain

GenBank accession no.	ID tree	Virus	Strain	Virus source	Year isolated	Country	No. haplotypes
AY996324	DUVV1	94286SA	DUVV	*Miniopterus* sp.	1981	South Africa	ND
DQ222422	R76	R76	EBLV1	*Eptesicus isabellinus*	1987	Spain	45
DQ222421	R75	R75	EBLV1	*E. isabellinus*	1989	Spain	48
DQ222419	155R99	155R99	EBLV1	*E. isabellinus*	1999	Spain	47
DQ222423	69R99	69R99	EBLV1	*E. isabellinus*	1999	Spain	46
DQ222424	80R99	80R99	EBLV1	*E. isabellinus*	1999	Spain	49
DQ222418	12418	12418	EBLV1	*E. isabellinus*	2000	Spain	46
DQ222420	69R00	69R00	EBLV1	*E. isabellinus*	2000	Spain	46
DQ222425	44R02	44R02	EBLV1	*E. isabellinus*	2002	Spain	50
HM212661	292R07	292R07	EBLV1	*E. isabellinus*	2007	Spain	51
HM212662	211R07	211R07	EBLV1	*E. isabellinus*	2007	Spain	46
HM212664	86R08	86R08	EBLV1	*E. isabellinus*	2008	Spain	46
HM212663	28458	28458	EBLV1	*E. isabellinus*	2009	Spain	46
AY062082	8268GR	9	EBLV1a	*E. serotinus*	1968	Germany	12
AY863348	4868GR	9395GER	EBLV1a	*E. serotinus*	1968	Germany	12
AY863350	5070GR	9398GER	EBLV1a	*E. serotinus*	1970	Germany	2
AY863351	5182GR	9399GER	EBLV1a	*E. serotinus*	1982	Germany	2
AY245845	4585DK	EBLV1a-DUV07	EBLV1a	*E. serotinus*	1985	Denmark	2
AY863349	4985GR	9396GER	EBLV1a	*E. serotinus*	1985	Germany	12
AY863369	6985PO	8615POL	EBLV1a	*E. serotinus*	1985	Poland	15
AY863371	7185RU	9397RUS	EBLV1a	*Homo sapiens*	1985	Russia	25
AY893368	6885HO	02022HOL	EBLV1a	*E. serotinus*	1985	Holland	2
AY863352	5286GR	9436GER	EBLV1a	*E. serotinus*	1986	Germany	9
AY863357	5786GR	9477GER	EBLV1a	*E. serotinus*	1986	Germany	13
AY863353	5387GR	9437GER	EBLV1a	*E. serotinus*	1987	Germany	6
AY863362	6287HO	9480HOL	EBLV1a	*E. serotinus*	1987	Holland	2
AY863372	7287UC	9443UKR	EBLV1a	*Vespertilio murinus*	1987	Ukraine	26
AY863373	7387DE	9479DEN	EBLV1a	*E. serotinus*	1987	Denmark	14
AY863374	7487DK	94110DEN	EBLV1a	*E. serotinus*	1987	Denmark	2
U89473	7387DK	94109DEN	EBLV1a	*E. serotinus*	1987	Denmark	2
U89476	7687HO	9474HOL	EBLV1a	*E. serotinus*	1987	Holland	17
AY863354	5488GR	9438GER	EBLV1a	*E. serotinus*	1988	Germany	2
AY863355	5589GR	9440GER	EBLV1a	*E. serotinus*	1989	Germany	8
AY863361	6189HO	9478HOL	EBLV1a	*E. serotinus*	1989	Holland	20
AY863363	6389HO	94116HOL	EBLV1a	*E. serotinus*	1989	Holland	2
U89461	6189GR	9439GER	EBLV1a	*E. serotinus*	1989	Germany	16
AY863356	5690GR	9441GER	EBLV1a	*E. serotinus*	1990	Germany	2
AY863358	5890GR	9481GER	EBLV1a	*E. serotinus*	1990	Germany	2
AY863370	7090PO	9394POL	EBLV1a	*E. serotinus*	1990	Poland	1
U89464	6490GR	9442GER	EBLV1a	*E. serotinus*	1990	Germany	2
AY863359	5992HO	9366GER	EBLV1a	*E. serotinus*	1992	Holland	3
AY863360	6092HO	9372HOL	EBLV1a	*E. serotinus*	1992	Holland	2
U89452	5292HO	9368HOL	EBLV1a	*E. serotinus*	1992	Holland	4
U89454	5493HO	9374HOL	EBLV1a	*E. serotinus*	1993	Holland	18
U89455	5594PO	96031POL	EBLV1a	*E. serotinus*	1994	Poland	5
AY863375	7595DK	02010DEN	EBLV1a	*E. serotinus*	1995	Denmark	2
AY863376	7697DK	02011DEN	EBLV1a	*E. serotinus*	1997	Denmark	7
AY863367	6798HO	02021HOL	EBLV1a	*E. serotinus*	1998	Holland	21
AY863366	6699HO	02020HOL	EBLV1a	*E. serotinus*	1999	Holland	19
AY863377	7799DK	02012DEN	EBLV1a	*E. serotinus*	1999	Denmark	2
AY863378	7899DK	02013DEN	EBLV1a	*E. serotinus*	1999	Denmark	2
AY863364	6400HO	02017HOL	EBLV1a	*E. serotinus*	2000	Holland	2
AY863365	6500HO	02018HOL	EBLV1a	*E. serotinus*	2000	Holland	20
AY863379	7900DK	02015DEN	EBLV1a	*E. serotinus*	2000	Denmark	11
AY863382	8201ES	01018SLO	EBLV1a	*E. serotinus*	2001	Slovenia	10
AY863380	8002DK	02016DEN	EBLV1a	*Ovis aries*	2002	Denmark	7
AY863381	8103FR	03002FRA	EBLV1a	*E. serotinus*	2003	France	24
AF124352	124352	34	EBLV1a	Unknown	Unknown	Unknown	23
AF124353	124353	EBL458861	EBLV1a	Unknown	Unknown	Unknown	22
AF124354	124354	RV627	EBLV1a	Unknown	Unknown	Unknown	14
AY062083	83XXGR	11	EBLV1a	Unknown	Unknown	Germany	2
AY062084	84XXDK	19	EBLV1a	Unknown	Unknown	Denmark	2
AY062085	85XXDK	20	EBLV1a	Unknown	Unknown	Denmark	7
AY062086	86XXDK	24	EBLV1a	Unknown	Unknown	Denmark	2
AY062087	87XXPO	66	EBLV1a	Unknown	Unknown	Poland	2
AY863393	9389FR	8919FRA	EBLV1b	*E. serotinus*	1989	France	41
AY863383	8392HO	9367HOL	EBLV1b	*E. serotinus*	1992	Holland	31
AY863386	8692HO	94113HOL	EBLV1b	*E. serotinus*	1992	Holland	32
AY863387	8792HO	94115HOL	EBLV1b	*E. serotinus*	1992	Holland	33
U89449	4992HO	9414HOL	EBLV1b	*E. serotinus*	1992	Holland	34
AY863384	8493HO	9376HOL	EBLV1b	*E. serotinus*	1993	Holland	33
AY863385	8593HO	9377HOL	EBLV1b	*E. serotinus*	1993	Holland	30
AY863391	9194SP	94285SPA	EBLV1b	*E. serotinus*	1994	Spain	45
AY863394	9495FR	9603FRA	EBLV1b	*E. serotinus*	1995	France	40
AY863395	9595FR	9906FRA	EBLV1b	*E. serotinus*	1995	France	39
AY245841	4197FR	113852	EBLV1b	*E. serotinus*	1997	France	27
AY863389	8997HO	02024HOL	EBLV1b	*E. serotinus*	1997	Holland	35
AY245844	4498FR	116883	EBLV1b	*E. serotinus*	1998	France	39
AY863388	8899HO	02019HOL	EBLV1b	*E. serotinus*	1999	Holland	33
AY245833	3300FR	121653	EBLV1b	*E. serotinus*	2000	France	28
AY245834	3400FR	132	EBLV1b	*E. serotinus*	2000	France	43
AY863396	9600FR	0001FRA	EBLV1b	*E. serotinus*	2000	France	43
AY863397	9700FR	0002FRA	EBLV1b	*E. serotinus*	2000	France	37
AY863398	9800FR	0003FRA	EBLV1b	*E. serotinus*	2000	France	38
AY863399	9900FR	0102FRA	EBLV1b	*E. serotinus*	2000	France	29
AY245837	3701FR	122319	EBLV1b	*E. serotinus*	2001	France	42
AY245842	4201FR	122154	EBLV1b	*E. serotinus*	2001	France	27
AY863400	0001FR	02031FRA	EBLV1b	*E. serotinus*	2001	France	42
AY863401	0101FR	02032FRA	EBLV1b	*E. serotinus*	2001	France	36
AY863402	0201FR	02033FRA	EBLV1b	*E. serotinus*	2001	France	27
AY245832	3202FR	123008	EBLV1b	*E. serotinus*	2002	France	44

*EBLV, European bat lyssavirus; ND, not determined.
